# Heart Failure and a Plant-Based Diet. A Case-Report and Literature Review

**DOI:** 10.3389/fnut.2019.00082

**Published:** 2019-06-11

**Authors:** Kathleen E. Allen, Divya Gumber, Robert J. Ostfeld

**Affiliations:** ^1^Department of Food and Nutrition, NewYork-Presbyterian, New York, NY, United States; ^2^Division of Cardiology, Montefiore Health System, Bronx, NY, United States

**Keywords:** diet, heart failure, hypertension, plant-based, vegetarian

## Abstract

A 54-year-old female with grade 3 obesity body mass index (BMI 45.2 kg/m^2^) and type II diabetes (hemoglobin A1c 8.1%) presented to her primary care physician in May 2017 with a chief complaint of left lower extremity edema. Work-up revealed heart failure with depressed left ventricular systolic function. Upon diagnosis, she substantially altered her lifestyle, changing her diet from a “healthy western” one to a whole food plant-based one. Guideline directed medical therapy for heart failure was also utilized. Over five and a half months, she lost 22.7 kg and reversed her diabetes without the use of diabetes medications. Her left ventricular systolic function normalized. Although causality cannot be determined, this case highlights the potential role of a plant-based diet in helping to reverse heart failure with reduced ejection fraction. This article will review how a minimally processed whole food plant-based dietary pattern and similar dietary patterns, such as the Dietary Approach to Stop Hypertension diet, may contribute to the reversal of left ventricular dysfunction.

## Case

A 54-year-old female with grade 3 obesity body mass index (BMI 45.2 kg/m^2^) and type II diabetes (hemoglobin A1c 8.1%) presented to her primary care physician in May 2017 with a chief complaint of left lower extremity edema. Venous duplex revealed no deep venous thrombosis and an X-Ray revealed lower extremity atherosclerosis with no fracture. She was sent to a cardiologist. Electrocardiogram demonstrated normal sinus rhythm and a left bundle branch block. Echocardiography revealed a left ventricular ejection fraction of 25% without significant valvular pathology; heart failure was diagnosed. Renal, liver, and thyroid function, as well as ferritin and potassium levels were within normal limits. HIV was non-reactive. She was not anemic. She was started on a beta-blocker, an ACE inhibitor, and a statin. Cardiac MRI in June 2017, revealed a dilated cardiomyopathy and an ejection fraction of 21%. Coronary CT angiogram revealed an Agatston coronary artery calcium score of 458. Extensive calcification on the CT angiogram precluded assessment of coronary artery stenosis. Hence cardiac catheterization was performed and revealed a cardiomyopathy out of proportion to coronary artery disease with a 30% proximal left anterior descending artery stenosis, a 25% proximal and a 60% distal left circumflex artery stenosis, and a 65% first obtuse marginal artery lesion. The left main and right coronary arteries were without stenosis. She was shaken by her diagnosis and became determined to adopt a more healthful diet. She changed her diet from “healthy western” to whole food plant-based (Table 1). She also started supplemental vitamin B12. She lost 22.7 kg in <6 months, resulting in a BMI of 35.1 kg/m^2^. Her diabetes resolved, with her hemoglobin A1c falling to 5.7% without the use of diabetes medications. Her baseline dyspnea on exertion improved considerably. Repeat echocardiography in November 2017 revealed a normal left ventricular ejection fraction of 55% (Table 2).

**Table 1:** Patient's dietary pattern pre- and post-dietary change.

**“Healthy western” diet**	**Plant-based diet (daily)**
• Chicken (without the skin) • Fish • Turkey • Eggs • Lean cuts of red meat • Small amounts of processed red meat • Diet soft drinks • Processed foods (e.g., chips and cakes) • 1–2 servings of vegetables or fruits daily	• *No* animal products • At least 3 servings dark leafy greens • At least 3 servings of vegetables • At least 3 servings of fruit • 1–3 servings of beans/legumes • 1–3 servings of whole grains • 1 Tablespoon herb/spice • 1 serving of raw unsalted nuts or seeds • 2 Tablespoons of hemp seeds/chia seeds/ground flax meal • At least one cup of tea/day • Limit packaged/processed foods

*Regarding the plant-based diet, patients are not given caloric or macronutrient goals and are invited to consume freely within these parameters*.

**Table 2:** Health parameters at baseline and after five and a half months on a plant-based diet.

**Parameter**	**Baseline**	**After 5 ½ months**
BMI	45.2 kg/m^2^	35.1 kg/m^2^
Hemoglobin A1c	8.1%	5.7%
Ejection fraction	25%	55%

Although causality cannot be determined, this case highlights the potential role of a plant-based diet in helping to reverse systolic dysfunction, or heart failure with reduced ejection fraction.

This article will review how a minimally processed whole food plant-based dietary pattern and similar dietary patterns, such as the Dietary Approach to Stop Hypertension (DASH) diet and vegetarian diet, may contribute to the reversal of left ventricular dysfunction. For the purposes of this case report and literature review, the term plant-based diet will include dietary patterns that are exclusively plant-based and dietary patterns that are predominantly plant-based, such as the DASH diet and vegetarian dietary patterns.

## Background

Heart failure (HF) is a condition in which the heart is unable to provide adequate blood flow to meet the normal metabolic needs of the body and can occur with either a reduced or a preserved left ventricular ejection fraction (1). HF is a leading cause of morbidity and mortality with a prevalence of more than 5.5 million in the US and 23 million globally (2). Each year in the US, over 550,000 individuals are newly diagnosed with HF (3)–about half die within 5 years (1).

Numerous risk factors for the development and progression of HF are influenced by diet, including inflammation, hypertension, dysbiotic microbiome, hyperlipidemia, obesity, and diabetes (4–6). However, the medical community has traditionally focused on pharmacotherapy and devices and not on nutrition in both the primary and secondary prevention of HF (7, 8).

This focus may occur because cardiologists receive little instruction on either nutrition or nutrition counseling (9, 10). In a recent survey of more than 900 cardiologists, although 95% believed that their role should include counseling patients about nutrition, 90% received minimal or no related training (10). This training deficit is not unique to cardiology and extends to most fields, including internal medicine and obstetrics/gynecology (9, 11, 12).

This deficit may represent a preventive opportunity lost throughout the lifecycle. The Barker Hypothesis suggests that the intrauterine environment influences cardiovascular health later in life (13, 14). In human and animal models, the presence of maternal obesity adversely impacted cardiac morphology and metabolism, predisposing offspring to cardiovascular disease (15, 16). Offspring of maternal pigs fed a high fat, high-calorie diet versus a standard diet have numerous structural and metabolic cardiac derangements that may put them at risk for HF (16). Human mothers consuming more meat and fish had offspring with elevated cortisol levels which may predispose to hypertension and the metabolic syndrome (17). Consequently, more healthful diets may provide both primordial prevention, and prevention throughout the lifecycle (18).

## Prospective Cohort Studies

Prospective cohort studies support the beneficial impact of plant-based dietary patterns on incident HF (19–23). In a study of 38,075 Finnish people over a median of 14.1 years, higher consumption of vegetables was associated with a lower incidence of HF in men, but not in women (21). Similarly, among 20,900 healthy male physicians in the Physicians' Health Study I, greater consumption of fruits and vegetables was associated with a decreased risk of HF (19). A subset of the Reasons for Geographic and Racial Differences in Stroke (REGARDS) Cohort of 15,569 persons with no Coronary Artery Disease or HF diagnosis was divided into five dietary patterns: Alcohol/Salads, Convenience, Plant-based, Southern, and Sweets. After a median follow-up of more than 7 years, patients with closer adherence to the Plant-based dietary pattern had lower risk of incident HF (23). In a prospective cohort from Sweden of 34,319 women without cardiovascular disease and cancer at initial assessment, after 12.9 years, greater fruit and vegetable consumption was associated with a lower rate of HF (22).

## The Influence of Diet on Heart Failure Risk Factors

### Inflammation

Elevated serum levels of inflammation increase risk factors for HF, as well as incident HF (24, 25). Plant-based diets (PBD) decrease serum levels of inflammation (26–29) and may be protective (5). This protective effect is in part mediated through Reactive Oxygen Species (ROS) or free radicals (30, 31).

ROS are unstable molecules that promote inflammation and may react with and damage myocardial cells leading to interstitial fibrosis, myocyte hypertrophy, and decreased myocardial contractility (32). PBDs with high levels of antioxidants and other bioactive compounds may be protective by reducing levels of ROS whereas animal-based diets with lower levels of antioxidants, may not (30, 33). In addition, the consumption of animal foods may facilitate ROS creation via heme iron (34) and other pro-inflammatory compounds such as N-Glycolylneuraminic acid (Neu5gc) (35), Advanced Glycation End-Products (AGEs) (36), trimethylamine amine (TMAO) (37), and nitrates utilized to preserve processed meats (38), thereby potentially further inducing myocardial dysfunction and HF (23, 24).

Moreover, vegetarian versus omnivorous diets are associated with lower plasma levels of the pro-oxidant compound, myeloperoxidase, which promotes atherosclerotic plaque formation, and progression. This promotion may lead to atherosclerotic plaque rupture, myocardial infarction, and HF (39).

### Hypertension

Hypertension (HTN) is a common precursor to HF, increasing its risk by 2–3 fold (40, 41). Accordingly, in the Framingham cohort, a blood pressure (BP) of ≥160/100 vs. <140/90 doubled the lifetime risk of incident HF (40). PBDs and predominantly PBDs, such as the DASH or vegetarian diet, may lower both systolic and diastolic BP (42–44), via a number of mechanisms, including favorably modifying the renin-angiotensin (45) and sympathetic nervous systems (46), greater potassium and decreased sodium consumption (47), improved blood vessel dilation (44, 46, 48), and changes in baroreceptors (46).

In prospective studies, red meat and poultry increased incident HTN (49) whereas plant proteins lowered it (50). In the Chicago Western Electric Study, greater consumption of fruits or vegetables was associated with significantly lower BP whereas beef, lamb, poultry, and veal consumption was associated with higher BP after 7 years (51). Similarly, in a large cohort from China of over 450,000 subjects, greater consumption of fresh fruit was associated with decreased systolic BP by 4 mmHg (52). The Diet Approach to Stop Hypertension (DASH) diet, which is low in animal products, total, and saturated fat and high in fruits and vegetables, reduced both systolic and diastolic BP by 5.5 and 3.0 mmHg, respectively, in hypertensive patients (53). Correspondingly, large prospective studies reported that greater adherence to a DASH diet was associated with a 22 and 37% decreased HF risk in men and women, respectively (54, 55). A PBD, however, may even further lower BP. A vegan diet has been associated with achieving a lower BP than omnivorous, pescatarian, and vegetarian diets (56).

### Microbiome

The gut microbiome consists of around one hundred trillion microorganisms and impacts our immunologic, cardiovascular, and overall health (57). For example, the pro-atherogenic compound, Trimethylamine N-oxide (TMAO) is formed, in part, via the interplay of the gut microbiome with the nutrients L-carnitine and choline (58). Increasing TMAO levels are associated with increasing severity of HF, rates of MI, and overall mortality (58–64). Accordingly, TMAO may induce vascular inflammation, increase platelet reactivity (58), and reduce reverse cholesterol transport (4). A PBD rapidly selects for a different microbiome than does an animal-based diet and hence produces less TMAO than does an animal-based diet (4) possibly accounting, in part, for the fewer cardiovascular events seen in those consuming a minimally processed plant-based diet (58, 65).

Furthermore, soluble dietary fiber, found exclusively in plant-based foods (66), nourishes healthful bacteria in the colon enabling them to produce short-chain fatty acids (67). Short-chain fatty acids then nourish the enterocyte (68) which may lead to reduced cholesterol biosynthesis by the enterocyte by down-regulating the enterocyte's expression of genes involved in cholesterol biosynthesis, and thereby lower serum cholesterol levels (69). As elevated serum cholesterol is a key cause of coronary artery disease and myocardial infarction (70–73), and hence HF, plant-based nutrition may further lower HF risk by impacting cholesterol biosynthesis in the enterocyte (4, 69).

### Hyperlipidemia

Plant-based diets lower serum lipid levels in part due to their soluble fiber, phytosterols, low saturated fat content, absence of cholesterol, and potentially due to their ability to reduce enterocyte cholesterol biosynthesis (69, 74, 75). Moreover, a PBD improves lipids levels specifically in patients with decreased left ventricular ejection fraction (76, 77). Conversely, animal-based diets are associated with increased serum cholesterol levels (78).

Furthermore, a PBD may render low-density lipoprotein (LDL) cholesterol particles less prone to oxidation (74, 79–81). Oxidized LDL particles are particularly atherogenic, damaging to endothelial cells, and promoting of inflammation and oxidative stress (82, 83).

While high-density lipoprotein (HDL) levels may decrease on a PBD (43), recent studies have suggested that increasing HDL levels may not be beneficial and may be harmful (84). In addition, it appears that a healthful lifestyle may render an HDL particle protective, while an unhealthful one renders it atherogenic (85). Accordingly, HDL efflux capacity, or its ability to perform reverse cholesterol transport, irrespective of the absolute HDL serum level, may be a more important measure of its potential beneficial health impact (43, 84, 86). Thus, aspects of a PBD, such as pistachios and almonds, may improve HDL efflux capacity (86, 87).

### Obesity

Obesity is a risk factor for HF and may account for approximately 11 and 14 percent of HF cases in men and women, respectively. Increases in BMI are linearly associated with an increase in HF risk, where those with obesity having twice the risk of HF compared to those with a healthy BMI (88). Similarly, in the Multi-Ethnic Study of Atherosclerosis (MESA) cohort, greater self-reported weight at 20 and 40 years of age was associated with increased HF risk later in life (89).

This increased risk may occur as obesity is associated with altered left ventricular remodeling (90). Furthermore, obesity may promote risk factors for HF, such as inflammation, HTN, dyslipidemia, DM, and sleep apnea (88, 91–93).

PBDs have been associated with greater weight loss (94, 95), decreased weight gain (96), and lower BMIs (97, 98) when compared with other dietary patterns. Conversely, greater intake of animal-based foods is correlated with a higher BMI (97, 98). Accordingly, a randomized controlled trial found a vegan diet to be associated with significantly greater weight loss (−7.5%) than a pesco-vegetarian, semi-vegetarian, or omnivorous diet (−3.2, −3.2, and −3.1%, respectively) over the course of 6 months (94). A prospective cohort study found each 250 g/d increase in meat (red meat, processed meat, and poultry) consumption to be associated with an increase in weight of 2 kg over 5 years (99). In the Seventh-Day Adventist study, mean BMI increased as diet became more animal-centric: 23.6 kg/m^2^ in vegans, 25.7 kg/m^2^ in lacto-ovo-vegetarian, 26.3 kg/m^2^ in pesco-vegetarians, 27.3 kg/m^2^ in semi-vegetarians, and 28.8 kg/m^2^ in non-vegetarians (98). PBDs likely contribute to the obtention and maintenance of a healthy weight through their high fiber content, low caloric density, and potentially, their ability to augment resting energy expenditure (100).

### Diabetes

Diabetes Mellitus (DM) is associated with an approximate 400% increase in the incidence of HF (101) and poorly controlled diabetes is associated with worse HF outcomes. Each 1% increase in hemoglobin A1c (HbA1c) was associated with a 12% increase in HF hospitalizations after controlling for other HF risk factors (102). Similarly, insulin resistance, often a precursor for diabetes, is associated with an increased risk of incident HF (103).

A PBD results in a lower incidence of diabetes (104, 105) and hemoglobin A1c levels (106, 107) and may reduce insulin requirements despite a lack of weight loss (108). As such, a PBD may improve HF incidence and outcomes (101, 102). As aforementioned, PBDs are associated with decreased inflammation (26–29), which may ameliorate diabetes by improving both insulin sensitivity and beta cell function (109, 110). Moreover, PBDs are low in saturated fat, which upon accumulation in muscle and hepatic cells, contributes to insulin resistance (100, 111, 112).

Accordingly, in the Seventh-Day Adventist Study, the relationship between Type 2 diabetes and degree of dietary plant consumption appeared to lie on a continuum, with a prevalence of: 2.9% in vegans, 3.2% in lacto-ovo vegetarians, 4.8% in pesco-vegetarian, 6.1% in semi-vegetarians, and 7.6% in non-vegetarians (98).

## Heart Failure With Preserved Ejection Fraction

Heart failure with preserved ejection fraction (HFpEF), or diastolic heart failure, is common and has similar symptoms and adverse outcomes as heart failure with reduced ejection fraction (113). It is hypothesized that HFpEF may be secondary, in part, to diffuse endothelial dysfunction, including that of the myocardial microvasculature, due partly, to increased inflammation (113). Many plant-based foods are anti-inflammatory (26), and increase Nitric Oxide bioavailability (114), thereby improving vascular health and potentially ameliorating this microvascular dysfunction (24, 113, 115). A PBD may also prevent or treat numerous comorbid conditions, such as obesity, HTN, kidney disease, and diabetes (44, 98, 104–107, 116–118), potentially providing additional preventive and therapeutic benefit. The Dietary Approach to Stop Hypertension in “Diastolic” Heart Failure (DASH—DHF) pilot study evaluated the impact of a DASH diet on 13 post-menopausal women with diastolic HF. After 3 weeks, consuming the DASH diet was associated with significant improvements in blood pressure, arterial elasticity, and oxidative stress (115).

## Conclusion

Our patient's improvements were temporally associated with her adoption of a whole food plant-based diet. Plant-based diets are associated with improvements in risk factors for heart failure and with direct benefits on cardiac metabolism and function. Given the burden of heart failure, its adverse prognosis, and the overall evidence to date, a plant-based diet should be considered as part of a multifaceted approach to heart failure care.

## Ethics Statement

Full name of the ethics committee: The Montefiore Einstein Institutional Review Board.

Consent Procedure: Written informed consent was obtained from the patient to publish her identifiable/case report data.

## Author Contributions

RO identified the case and had sole access to patient information. RO, KA, and DG all performed background research, and contributed both to the writing and editing of the paper.

### Conflict of Interest Statement

RO consulted for Better Therapeutics. The remaining authors declare that the research was conducted in the absence of any commercial or financial relationships that could be construed as a potential conflict of interest.

## Abbreviations

BMI: Body mass index
DASH: Dietary Approach to Stop Hypertension
HDL: High density lipoprotein
HF: Heart Failure
HFpEF: heart failure with preserved ejection fraction
HTN: Hypertension
LDL: Low-density lipoprotein
PBD: Plant-based Diet.
